# Dermatology resident comfort level treating hair conditions related to patients with skin of color

**DOI:** 10.1097/JW9.0000000000000137

**Published:** 2024-06-12

**Authors:** Starling Tolliver, Camilla Cascardo, Nikita Wong, Yasmine Abushukur, Geoffrey Potts

**Affiliations:** a Department of Dermatology, Wayne State University, Detroit, Michigan; b Oakland University William Beaumont School of Medicine, Rochester, Michigan; c Wayne State University School of Medicine, Detroit, Michigan

**Keywords:** Afro-textured hair, general dermatology, hair care, hair loss, resident education, skin of color

## Abstract

**Background:**

Although recent studies demonstrated resident satisfaction in the treatment of skin of color (SOC) related disease, comfort levels treating hair specific to populations within the SOC spectrum is unclear.

**Objective:**

The purpose of this study is to assess dermatology residents comfort level in recognizing and treating various common hair conditions with a focus on those specific to SOC.

**Methods:**

An Institutional Review Board-approved survey was distributed to United States residents of Accreditation Council for Graduate Medical Education-accredited dermatology programs. Data pertaining to hair care knowledge and treatment comfort levels were collected. Analysis was completed using equal variance 2-sample *t* tests and analysis of variance *F* tests, *P* < .05.

**Results:**

Dermatology residents were relatively comfortable with common conditions such as androgenetic alopecia and alopecia areata, but uncomfortable with creating healthy hair regimens, discussing natural hair care products, and treating trichorrhexis nodosa. Resident self-identification as underrepresented in medicine significantly impacted resident knowledge of hair care and treatment in patients with SOC.

**Limitations:**

This study was limited due to small sample size and potential recall bias.

**Conclusion:**

This study highlights knowledge gaps in understanding hair-related care for patients with SOC, affirming the continued importance of diversifying dermatology programs as well as hair-specific training for residents.

**What is known about this subject in regard to women and their families?**

- Black women experience hair loss and difficulty with hair growth retention at higher rates than most races.
- Black women have disproportionate effects given complex hair structure, genetic factors, and cultural practices within the community.
- Most Black women do not seek dermatologic care when experiencing issues related to hair.
- Studies have suggested that this is due to lack of experience of the physician when dealing with afro-textured hair as well as lack of patient trust.
- There are studies on increasing knowledge regarding skin of color (SOC) in resident education, however, to our knowledge, no studies have focused on resident comfort level in treating SOC-specific hair disease in this community.

**What is new from this article as messages for women and their families?**

- SOC education has been a hot topic over recent years and focus on increasing knowledge in this area has exploded.
- Many of these initiatives have focused solely on topics related to skin, however, specifically afro-textured hair remains an underserved and under-researched entity.
- Many dermatology residents express satisfaction with knowledge of SOC-related conditions, but comfort levels remain unclear relating to diagnosing, treating, and counseling on SOC hair conditions in Black women.
- Our article provides evidence of a disparity in resident education regarding the management of hair conditions specific to this community.
- It also recognizes differences in comfort level with these conditions when accounting for underrepresented background.

## Introduction

Nearly half of the US population will be made up of individuals with skin of color (SOC) by the end of the century.^1,2^ Despite the rapid increase in this population, coverage of issues pertinent to SOC, especially those of those of African ancestry in dermatologic educational resources, remains variable. In addition, much of the current literature emphasizes disease processes related to skin alone.^3^ Research is limited on the prevalence of characteristic hair disease in patients with afro-textured hair compared to patients with non-afro-textured hair, however, studies have shown high rates of breakage, traction alopecia (TA), central centrifugal cicatricial alopecia in Black women.^4,5^ As many as 50 to 90% of Black women experience hair loss^6,7^ and they often have increased morbidity such as severe permanent scarring and deep psychological impact associated with these illnesses.^8^

Despite recent studies demonstrating a majority of dermatology residents being satisfied with their knowledge of disease primarily seen in patients with SOC,^9^ many patients with SOC attribute their dissatisfaction with their dermatologic care due to the inadequate training of dermatologists on these conditions.^10^ Moreover, to our knowledge no studies have focused on hair alone. This underscores the importance of exploring and understanding the gaps present in dermatology resident training as it pertains to individuals of varying hair types. Together, this signifies an underlying importance of both skin and hair mastery in all skin types. This study intends to assess dermatology residents’ level of knowledge and comfort treating various hair-related conditions and those specific to SOC patients.

## Methods

### Data collection

An institutional review board-approved Qualtrics survey was generated and distributed via e-mail to the Association of Professors of Dermatology membership list on February 24, 2022. Program directors and academic dermatologists were encouraged to distribute the survey to residents within their institution. Participation eligibility was limited to residents of an Accreditation Council for Graduate Medical Education-accredited US dermatology residency program. All residents consented to participation prior to providing responses.

Hair-related conditions assessed included, tinea capitis, TA, alopecia areata, androgenetic alopecia, lichen planopilaris, frontal fibrosing alopecia, central centrifugal cicatricial alopecia (CCCA), discoid lupus erythematosus, dissecting cellulitis, folliculitis decalvans, trichotillomania, and trichorrhexis nodosa (TN). Residents reported how frequently they treated patients with each of these conditions. Frequencies ranged from 1 to 6 respectively, (never or 1 time per year, once every 6–12 months, once a month to once every 6 months, once a week to once a month, every day to once a week, or multiple times per day). Residents were asked to rank their comfort level with recognizing and treating each of these conditions. Comfort levels ranged from 1 to 5 respectively (extremely uncomfortable, somewhat uncomfortable, neither comfortable nor uncomfortable, somewhat comfortable, or extremely comfortable). Using the same scale, residents were asked to describe their comfort level with understanding the basic hair morphology of SOC patients as well as their comfort counseling individuals with SOC on proper hair practices, best hair products, and healthy hair regimens. Demographic data pertaining to residents’ dermatology program, residency location (reported as state and coded by geographic region), year of residency (PGY-2, PGY-3, and PGY-4), clinical setting (urban, suburban, rural, and mixed), and access to a hair-specific clinic within each residency program were collected. Residents were also asked whether or not they identify as underrepresented in medicine and various clinical interests.

### Statistical analysis

Responses were extracted using Microsoft Excel and coded within a spreadsheet before statistical analyses were run using Windows SAS/STAT software (Version 9.4, SAS Institute Inc., Cary, NC). Summary statistics were calculated for all available data. Equal variance 2-sample *t* tests were used to compare the means for numerical variables of normally distributed data. Analysis of variance tests were run when comparing resident comfort treating various hair-related conditions in the general population versus in SOC patients. Results were considered statistically significant if *P* < .05. Qualitative analysis was conducted through open-ended questions. The Wayne State University Institutional Review Board deemed this study exempt.

## Results

A total of 45 US-accredited dermatology programs across 146 residency total programs (30% response rate) were included in our analysis. Each class was represented fairly equally with varied geographical locations and practice types. Our study reported 24% of participants from underrepresented backgrounds and 10.7% of the total residents had an interest in SOC. Of the 121 total residents in our survey, 45.5% noted a hair-specific clinic at their residency program (Supplementary Table S1, http://links.lww.com/IJWD/A42). The residents were asked to report how often they observed each hair condition, their comfort recognizing each, and their comfort level treating each. Residents reported seeing most hair conditions at least once a month to once every 6 months except for folliculitis decalvans, trichotillomania, and TN (2, 2, and 2, respectively). When comparing all non-SOC hair-related conditions residents felt overall “either somewhat comfortable” or “extremely comfortable” regarding recognition and treatment of these diseases (Supplementary Table S2, http://links.lww.com/IJWD/A43).

When considering comfort with afro-textured hair conditions dermatology residents as a whole felt “neither comfortable nor uncomfortable” or “somewhat uncomfortable” (Table 1). Resident comfort in counseling patients on protective hair practices differed significantly depending on their background (*P* = .0034), as did their comfort in counseling patients on hair product recommendations for natural hair care (*P* = .0006). Comfort level also differed significantly in relation to understanding the basic science of hair morphology in SOC individuals and its unique characteristics (*P* = .0011) and their comfort developing a healthy hair regimen (*P* = .0016) with residents underrepresented in medicine having higher degrees of comfort. At the same time, however, the reported comfort levels were respectively “neither comfortable nor somewhat comfortable” versus “somewhat uncomfortable” in all categories except counseling on protective styles and prescribing treatments for TA. Here, residents felt “somewhat comfortable” versus “neither comfortable nor somewhat comfortable.” There was no significant difference in comfort prescribing treatment regimens for CCCA and TN (Table 2). Resident comfort with treating hair conditions did not differ significantly by geographical region or by clinic setting. Residents participating in a program with access to a hair-specific clinic felt more comfortable counseling patients on protective hair practices (*P* = .0298), however, there was no difference in comfort level counseling on specific product use or in the development of healthy hair regimens.

**Table 1:** Overall resident comfort with afro-textured hair conditions

How comfortable do you feel counseling on protective hair practices in afro-textured hair?	
*N*	121
Mean (SD)	3 (1.2)
Median	3
How comfortable do you feel counseling on hair product use for natural hair care in Black patients?	
*N*	121
Mean (SD)	3 (1.3)
Median	2
How well do you feel you understand the basic science of hair morphology in Black individuals as well as its unique characteristics?	
*N*	121
Mean (SD)	3 (1.2)
Median	2
How comfortable do you feel developing a healthy hair regimen for Black patients?	
*N*	121
Mean (SD)	3 (1.2)
Median	2
How comfortable do you feel in prescribing treatment regimens for CCCA in Black patients?	
*N*	121
Mean (SD)	3 (1.2)
Median	3
How comfortable do you feel in prescribing treatment regimens for traction alopecia in Black patients?	
*N*	121
Mean (SD)	3 (1.2)
Median	4
How comfortable do you feel in prescribing treatment regimens for trichorrhexis nodosa in Black patients?	
*N*	121
Mean (SD)	2 (1.2)
Median	2

CCCA, central centrifugal cicatricial alopecia.

**Table 2:** Resident comfort treating afro-textured hair conditions given underrepresented background

	Do you come from a background underrepresented in medicine?		*P*
	No (*N* = 92)	Yes (*N* = 29)	
How comfortable do you feel counseling on protective hair practices in afro-textured hair? (Scale 1–5)			.0034^a^
*N*	92	29	
Mean (SD)	3 (1.2)	4 (1.1)	
Median	3	4	
How comfortable do you feel counseling on hair product use for natural hair care in Black patients? (Scale 1–5)			.0006^a^
*N*	92	29	
Mean (SD)	2 (1.2)	3 (1.3)	
Median	2	3	
How well do you feel you understand the basic science of hair morphology in Black individuals as well as its unique characteristics? (Scale 1–5)			.0011^a^
*N*	92	29	
Mean (SD)	2 (1.1)	3 (1.3)	
Median	2	3	
How comfortable do you feel developing a healthy hair regimen for Black patients? (Scale 1–5)			.0016^a^
*N*	92	29	
Mean (SD)	2 (1.2)	3 (1.2)	
Median	2	3	
How comfortable do you feel in prescribing treatment regimens for CCCA in Black patients? (Scale 1–5)			.0666^a^
*N*	92	29	
Mean (SD)	3 (1.2)	4 (1.2)	
Median	3	4	
How comfortable do you feel in prescribing treatment regimens for traction alopecia in Black patients? (Scale 1–5)			.0415^a^
*N*	92	29	
Mean (SD)	3 (1.3)	4 (1.1)	
Median	3	4	
How comfortable do you feel in prescribing treatment regimens for trichorrhexis nodosa in Black patients? (Scale 1–5)			.0677^a^
*N*	92	29	
Mean (SD)	2 (1.2)	3 (1.4)	
Median	2	2	

CCCA, central centrifugal cicatricial alopecia.

a Equal variance 2-sample *t* test.

## Discussion

Our study assessed overall dermatology resident comfort level in recognizing, counseling, and treating common hair conditions compared to afro-textured hair-related conditions. CCCA, TN, and TA are hair diseases that disproportionately affect the Black community. This is due to a combination of factors including genetic and environmental causes that can lead to significant scarring and psychological distress. Early recognition and treatment are vital to control disease progression and hair regrowth. To our knowledge, this analysis is the first of its kind assessing the degree of disparity within hair-related education in dermatology residency. Even further, there is a paucity of literature reflecting the true nature of hair disparities in patients with SOC. Some studies have shown differences in clinical trial enrollment for and disease presentation of alopecia areata, toxic exposure to chemicals in hair products, physical activity due to hair styling, advocacy efforts, and utilization of dermatologists for cicatricial alopecias.^11–16^ Given that underrespresented in medicne residents tended to feel more comfortable managing specific hair conditions, the true level of resident comfort with managing these conditions may actually be overestimated as they represented 24% of participants. Even though 45.5% of participants reported having a hair-specific clinic, the majority of responses from this group showed no differences in comfort level treating diseases like CCCA, TA, and TN. This suggests that having a hair clinic alone may not confer adequate benefit to hair-specific education other than by increased comfort in counseling.

Comfort levels in treatment of CCCA and TN were statistically insignificant, although treatment of CCCA was reported as “somewhat comfortable” by the underrepresented in medicine group. The authors believe that training on the recognition and treatment of CCCA with recent advancements in understanding of this disease process may attribute to this. As for TN, there has been little progression in the understanding and treatment of TN, or hair breakage, especially in Black women. Therefore, both groups noting “somewhat uncomfortable” levels is reasonable as many Black women with TN typically do not seek care from a dermatologist due to the patient’s perceived lack of foundational knowledge of physicians in treating these conditions as well as adequate outside resources.^6^ To combat this, Gorbatenko-Roth et al. suggest several measures that may improve SOC patient dermatologic care satisfaction. These include enhancing residency training in SOC, increasing cultural competency, cost-conscious care, and empathetic communication skills.^10,17^ Forming alliances with hair stylists with knowledge of protective hair care practices could also improve patient care and trust in the community.

While most dermatology resident participants feel at least somewhat comfortable recognizing and treating a variety of hair conditions overall, comfort levels in managing afro-textured hair-specific concerns is limited demonstrating a disparity in comfort level regarding SOC-related hair conditions regardless of background. Overall lack of comfort may be linked to a lack of direct ongoing patient care experience with this population, less focused hair care curriculums, general lack of interest, or absence of evidence-based strategies for intervention. Although there have been many calls to action for increasing SOC in dermatology curricula over the last 10 years, few have focused broadly on hair-related issues in this specific population.^18–20^ Improving residents’ comfort level of counseling in this community can allow patients to feel understood, thus increasing the likelihood of following recommendations, returning for follow-up care, and potentially improving the physical and psychological health of patients.^21^ It is the author’s opinion that it is important that increased focus is placed on hair issues in this community, not just CCCA, as many of the prior studies related to SOC education narrowly focused on hair as a part of the full conversation.^9,10,17–20^

Of note, residents from underrepresented backgrounds were more comfortable counseling on multiple aspects of healthy hair care likely due to higher community awareness of specific hair disorders and increased interest in healthy hair strategies for these common conditions. In the past, these attributes were not typically valued as highly as other metrics within the residency selection process. However, more recently, the field of dermatology has mitigated this problem through holistic review and diversity initiatives.^17^ Although improvements have been made, the field continues to lag when it comes to diversity in both residents and faculty of color.^17,22^ Thus, it is important to continue to strive toward increasing diversity within academic faculty and trainees, which may also aid in improving collective knowledge through cross-cultural sharing in treating patients with SOC.

Future studies are necessary to assess the extent of disparities related to afro-textured hair and potential curriculum interventions to assist with improving comfort level of managing and counseling in these patients.

## Limitations

Limitations of this study include its small sample size, potential lack of generalizability, potential recall bias inherent to survey data collection methods, and selection bias of those who chose to complete the survey. Additionally, we realize that increased comfort treating a condition does not necessarily mean all aspects of the condition are being appropriately treated. Nonetheless, the purpose of this study is to address dermatology resident comfort counseling on safe hair practices amongst SOC patients in clinical settings, comfort level treating these conditions and to shed light on a topic warranting further investigation.

## Conclusion

To our knowledge, this is the first study examining dermatology resident comfort level in recognizing and treating various hair disorders, especially those specific to SOC. Dermatology residents can better serve their patients by being more comfortable counseling SOC hair-related concerns. Integrating diverse learning opportunities into resident curricula regarding the presentation of hair conditions in SOC patients may improve resident confidence in clinical settings. Additionally, we believe striving for greater diversity in dermatology residencies can help mitigate this particular gap in patient care through sharing of information and patient comfort. More studies are needed to assess resident comfort treating SOC hair-related conditions. Dermatology residency programs need to continually evaluate the degree of exposure their residents receive to SOC hair-related conditions within their respective curriculums and clinical settings.

## Conflicts of interest

None.

## Funding

Supported by the Wayne State University Graduate Medical Education Seed Grant.

## Study approval

The authors confirm that any aspect of the work covered in this manuscript that has involved human patients has been conducted with the ethical approval of all relevant bodies: Wayne State University IRB approval #21-08-3933.

## Author contributions

ST, CC, NW, YA, and GP: Study conception and design. ST and CC: Data collection. ST, CC, NW, YA, and GP: Analysis and interpretation of results. ST, CC, NW, YA, and GP: Draft manuscript preparation. All authors reviewed the results and approved the final version of the manuscript.

## Supplementary data

Supplementary material associated with this article can be found at http://links.lww.com/IJWD/A42 and http://links.lww.com/IJWD/A43.

## Supplementary Material
