# Supplementary material for: Dermatology resident comfort level treating hair conditions related to patients with skin of color
Source: Int J Womens Dermatol. 2024 Jun 12;10(2):e137. doi: 10.1097/JW9.0000000000000137 (PMC11168838; doi:10.1097/JW9.0000000000000137)
Supplement: Supplementary file 1 [file jw9-10-e137-s001.pdf]

Supplementary Table 1: Demographic Data of Survey Respondents (n=121)

**Residency Year**	**Percentage**
PGY-2	35.5%
PGY-3	32.2%
PGY-4	32.2%
**Program Geographical Region in United States**	
Southeast	11.6%
Midwest	22.3%
Northeast	19.0%
West	18.2%
Southwest	11.6%
**Do you come from a background underrepresented in medicine?**	
No	76.0%
Yes	24.0%
**Dermatology Practice Setting**	
Urban	47.9%
Mixed	24.0%
Suburban	23.1%
Rural	5.0%
**Clinical Interests**	
General Dermatology	62.8%
Procedural/Surgical	25.6%
Oncological Dermatology	10.7%
Skin of Color	10.7%
Complex Medical Dermatology	8.3%
Cosmetics	8.3%
Specific Condition Interest	8.3%
Pediatric Dermatology	6.6%
Dermatopathology	6.6%
Hair Loss	2.5%
**Hair-Specific Clinic**	
No	64.5%
Yes	45.5%
