# Supplementary material for: Dermatology resident comfort level treating hair conditions related to patients with skin of color
Source: Int J Womens Dermatol. 2024 Jun 12;10(2):e137. doi: 10.1097/JW9.0000000000000137 (PMC11168838; doi:10.1097/JW9.0000000000000137)
Supplement: Supplementary file 2 [file jw9-10-e137-s002.pdf]

Supplementary Table 2: Observation Frequency, Recognition Comfort, and Treatment Comfort by Hair Condition (n=121)

**How *often* do you see ____?**	**Mean (SD)**	**Median**	**How comfortable do you feel *recognizing* ____?**	**Mean (SD)**	**Median**	**How comfortable do you feel *treating* ___?**	**Mean (SD)**	**Median**
**TC**	3 (1.0)	3.0	**TC**	4 (0.8)	4.0	**TC**	4 (0.8)	4.0
**TA**	3 (1.0)	3.0	**TA**	4 (0.8)	4.0	**TA**	4 (0.9)	4.0
**AlA**	4 (0.8)	4.0	**AlA**	5 (0.6)	5.0	**AlA**	5 (0.6)	5.0
**AGA**	5 (0.8)	5.0	**AGA**	5 (0.5)	5.0	**AGA**	5 (0.6)	5.0
**LPP**	3 (1.0)	3.0	**LPP**	4 (0.8)	4.0	**LPP**	4 (0.9)	4.0
**FFA**	3 (1.0)	3.0	**FFA**	4 (0.9)	4.0	**FFA**	4 (0.9)	4.0
**CCCA**	3.0 (1.2)	3.0	**CCCA**	4 (0.9)	4.0	**CCCA**	4 (0.9)	4.0
**DLE**	3.0 (0.9)	3.0	**DLE**	4 (0.8)	4.0	**DLE**	4 (1.0)	4.0
**DC**	3 (0.9)	3.0	**DC**	4 (1.0)	4.0	**DC**	3 (1.0)	4.0
**FD**	2 (0.9)	2.0	**FD**	3 (1.0)	4.0	**FD**	3 (1.0)	3.0
**TTM**	2 (0.8)	2.0	**TTM**	4 (0.9)	4.0	**TTM**	3 (1.0)	3.0
**TN**	2 (0.9)	1.0	**TN**	3 (1.0)	2.0	**TN**	2 (1.0)	2.0

Tinea capitis (TC), traction alopecia (TA), alopecia areata (AlA), androgenetic alopecia (AGA), lichen planopilaris (LPP), frontal fibrosing alopecia (FFA), central centrifugal cicatricial alopecia (CCCA), discoid lupus erythematosus (DLE), dissecting cellulitis (DC), folliculitis decalvans (FD), trichotillomania (TTM), and trichorrhexis nodosa (TN)
